# Values and value-related responses in early adolescence: evidence from two large student surveys in Malta

**DOI:** 10.3389/fpsyg.2026.1923199

**Published:** 2026-09-17

**Authors:** Lucianne Zammit, Joseph Giordmaina, Vincent Marmara, Christine Fenech, May Agius, Roger Tirazona

**Affiliations:** 1 Department of Education Studies, Faculty of Education, University of Malta, Msida, Malta; 2 Department of Business & Enterprise Management, Faculty of Economics, Management & Accountancy, University of Malta, Msida, Malta; 3 Department of Human Communication Sciences and Disorders, Faculty of Health Sciences, University of Malta, Msida, Malta

**Keywords:** adolescent values, digital responsibility, early adolescence, item-level survey, Malta, pro-social principles, school belonging, social cohesion

## Abstract

**Background:**

Early adolescence is an important period for value development, yet large-scale values research has focused mainly on adults or older adolescents. Evidence concerning younger adolescents, particularly in small-state contexts, remains limited.

**Objective:**

This study examined item-level patterns of value endorsement and related responses by Year 7 and Year 8 students in Malta. It also explored differences by gender, school sector, country of birth and religious affiliation.

**Methods:**

Two age-appropriate cross-sectional surveys were completed by 718 Year 7 students and 615 Year 8 students through voluntary, school-mediated samples. Responses were analyzed at item level using descriptive and exploratory inferential analyses, with effect sizes used to assess the magnitude of subgroup differences.

**Results:**

Value-importance and normative-attitude items generally elicited strong support for prosocial principles. Responses were more varied on some self-reported behavior and scenario-based items, particularly those concerning admitting mistakes, challenging unfair treatment and responding to online harm. Gender was the most consistent subgroup correlate, although reported effects were small or very small and response distributions overlapped substantially.

**Conclusion:**

The findings describe favorable responses to many prosocial principles among the participating students and identify contexts in which reported action or intended action was less uniform. They do not establish nationally representative prevalence, equivalent latent value structures across demographic groups, or a direct relationship between values and observed behavior.

## 1 Introduction

Values are broad principles through which individuals evaluate what is important and desirable in life. Schwartz conceptualizes values as desirable, trans-situational goals that vary in importance and serve as guiding principles in people’s lives (Schwartz, 1992, 1994, 2012). Unlike attitudes, which concern evaluations of particular objects or situations, values represent underlying motivational goals that may influence how individuals interpret situations, establish priorities and select courses of action. Their relationship with behavior is not automatic, however, because the expression of a value may depend on the circumstances, the presence of competing motivations and the opportunities for action available to the individual (Bardi and Schwartz, 2003; Sagiv et al., 2017).

Schwartz’s model organizes basic human values within a motivational continuum. Values situated close to one another express compatible motivations, whereas values on opposing sides of the continuum may come into conflict. At a higher level, self-transcendence values emphasize concern for others and acceptance of difference, while self-enhancement values prioritize achievement, status and personal advantage. Openness-to-change values emphasize independence and readiness for new experiences, whereas conservation values relate to security, conformity and tradition (Schwartz, 1992, 2012; Schwartz et al., 2012). In the present study, this theory supplies a conceptual vocabulary for distinguishing values from adjacent constructs; it is not treated as a measurement model because the questionnaires do not contain the items required to reproduce or test the Schwartz value structure.

Although values show a degree of continuity, they are not fully fixed before adolescence. Developmental research indicates that children can distinguish among the motivational meanings represented in Schwartz’s model, while the organization and relative importance of values may continue to change during childhood and adolescence (Cieciuch et al., 2016; Daniel et al., 2023). Longitudinal studies of early adolescence have similarly identified substantial stability in individual differences alongside mean-level changes in the importance assigned to particular values (Daniel and Benish-Weisman, 2019; Vecchione et al., 2020). Adolescent value development should consequently not be understood as a general rise or decline in morality. It may instead involve changes in the relative priority assigned to different values, changes in the ordering of individuals within a group, or changes in the internal organization of the value system.

The measurement of values among children and adolescents requires developmentally appropriate methods. Values are abstract constructs, and instruments designed for adults may contain concepts or life situations that are not equally meaningful to younger respondents. The Picture-Based Value Survey for Children, for example, translates the motivational content of Schwartz’s values into concrete situations presented through images (Döring et al., 2010). Subsequent studies have continued to use age-adapted approaches to examine children’s value priorities and value-expressive behavior (Scholz-Kuhn et al., 2023).

Research examining adolescents’ own accounts also supports the use of accessible and context-sensitive approaches. Lewis-Smith et al. (2021) found that adolescents aged 12–17 could describe the meanings of their values and connect them to identity, relationships, decision-making and preferred ways of living. Such findings indicate that young people do not necessarily experience values only as abstract moral principles. Values may also function as personally meaningful reference points through which they interpret everyday relationships, choices and future aspirations. Instruments developed for younger respondents need not reproduce adult questionnaires directly, although researchers must specify what their instruments measure and should not claim equivalence with established scales without appropriate validation.

Having clarified both the developmental status and the measurement of values, the next question concerns the settings in which value-related judgments and practices occur. Early adolescence expands young people’s participation in peer, school and digital environments, making these contexts central to interpretation.

Early adolescence is especially important because it is characterized by changes in self-understanding, autonomy and participation in family, peer and institutional relationships (Steinberg and Morris, 2001; Smetana et al., 2006). These developments alter the contexts in which young people make judgments and decisions. Values during adolescence are therefore expressed within relationships and social institutions rather than operating as isolated psychological characteristics.

Peer relationships become particularly salient during this period. They can provide support, companionship, recognition and opportunities for cooperation, but peer groups also establish norms concerning acceptance and appropriate conduct (Steinberg and Morris, 2001; Smetana et al., 2006). Peer influence is not inherently negative: its effects depend on the norms and practices of the group, and peers may encourage prosocial as well as harmful conduct. Nevertheless, the growing importance of friendships makes cooperation, loyalty, trust, inclusion and belonging especially relevant contexts in which adolescent values may be expressed.

Values and peer relationships may also influence one another. Kindschi et al. (2019) found that similarities in adolescents’ value priorities were associated with both friendship selection and social influence. Adolescents may be more likely to form relationships with peers whose value priorities resemble their own, while interaction within friendship groups may contribute to increasing similarity. Values should therefore be understood as personal orientations that are embedded in social relationships rather than as characteristics formed and expressed independently of them.

The relationship between values and behavior nevertheless requires careful interpretation. Bardi and Schwartz (2003) found systematic associations between values and corresponding behaviors, but the strength of these relationships differed across values. The relevance of a value to a particular situation, the presence of competing priorities and the opportunities or constraints within the immediate context may all influence whether a value is enacted. Endorsement of fairness, honesty or inclusion cannot therefore be treated as evidence that an individual will act in accordance with that value in every situation.

The relationship may also be reciprocal. Longitudinal evidence indicates that values can influence behavior while repeated behavior and experience may contribute to changes in value priorities (Vecchione et al., 2016). Associations have also been identified between children’s personal values and teacher-rated classroom behavior, although these relationships are neither simple nor uniform (Scholz-Kuhn et al., 2023). Distinguishing between reported values and reported behavior does not mean that the two are unrelated; rather, it means that one cannot be inferred directly from the other.

The interpersonal processes described above also have a wider societal relevance. When patterns of trust, inclusion and cooperation extend beyond individual relationships, they may bear on social cohesion; however, personal value endorsement and societal cohesion remain analytically distinct.

The significance of adolescent values extends beyond individual development. Values such as respect, fairness, trust, inclusion, cooperation and responsibility may contribute to social cohesion, although their endorsement should not be treated as a direct measure of cohesion. Schiefer and van der Noll (2017) identify three recurring dimensions of social cohesion: the quality of social relations, identification with or belonging to a social entity, and orientation toward the common good. They distinguish these dimensions from the conditions that may promote cohesion and the outcomes that may result from it.

Reported values may therefore provide evidence about principles capable of supporting constructive relationships, inclusive belonging and concern beyond immediate self-interest, but they cannot establish that a school or society is cohesive. Respect, fairness and trust are relevant to the quality of social relations; inclusion and recognition are relevant to belonging; and cooperation and responsibility are relevant to orientation toward the common good (Schiefer and van der Noll, 2017). These concepts are related but not equivalent.

Belonging is particularly significant within students’ educational and social experience. A meta-analysis of 82 studies in secondary education found that school belonging was associated with motivational, social–emotional, behavioral and academic outcomes, although the strength of the relationships varied across domains (Korpershoek et al., 2020). Belonging should not itself be treated as synonymous with a personal value, but its importance to students’ wellbeing and participation makes it a relevant context in which values are expressed.

Belonging may also differ in breadth and inclusiveness. Attachment to family, friendship groups, schools and local communities can support care and participation, but strong identification with a close group does not necessarily extend to unfamiliar others. Abela’s (2004) analysis of solidarity in Malta and Europe distinguishes forms of solidarity centered on close interpersonal relationships from broader orientations extending beyond immediate social circles. This distinction is relevant to social cohesion because cohesive living requires not only attachment within familiar groups but also openness toward difference and participation across group boundaries.

Schools provide an important context for values research because they combine formal teaching with sustained interpersonal and institutional experience. Students encounter curricular messages concerning values, citizenship and responsibility, but they also experience relationships with teachers and peers, institutional rules, disciplinary procedures and opportunities for participation. Values are therefore communicated not only through what schools explicitly teach but also through how school life is organized and experienced.

The OECD (2021) describes values and attitudes as being explicitly taught, “caught” through relationships and institutional practices, “sought” through reflection and inquiry, and embodied in educational aspirations. This formulation highlights the limitations of treating values education as the communication of definitions or prescribed principles. A school may teach fairness formally, but students’ understanding of fairness may also be shaped by whether institutional procedures appear transparent and consistent. Respect may likewise be discussed in the curriculum while being reinforced or contradicted through everyday interaction.

School climate offers a framework for understanding these wider experiences. Cohen et al. (2009) describe school climate as encompassing safety, relationships, teaching and learning, and the institutional environment. School climate is conceptually distinct from students’ personal values: the former concerns shared perceptions and experiences of the school environment, while the latter concern personal motivational and normative orientations. Nevertheless, the two may be related because school relationships and institutional practices provide contexts in which values are communicated, interpreted and enacted. Research on value development in educational settings has consequently emphasized the interaction between personal values and school context (Makarova et al., 2025).

School environments are also relevant to inclusion and belonging. Demographic diversity does not automatically produce positive intergroup relationships; its implications depend partly on whether schools create conditions that support equitable participation and constructive interaction (Nishina et al., 2019). Research concerning students with immigrant backgrounds has similarly identified associations between an immigrant-inclusive school climate, multicultural education and school belonging (Choi et al., 2025). Although these findings should not be generalized to every population or form of difference, they support the proposition that inclusion and belonging are partly shaped by students’ educational environments.

Digital communication provides an additional context in which adolescents encounter questions of respect, responsibility, privacy and participation. Digital citizenship has sometimes been understood narrowly in terms of technological access or competence, but Choi’s (2016) concept analysis identified four broader dimensions: ethics, media and information literacy, participation or engagement, and critical resistance. Digital citizenship therefore involves normative judgment and responsible participation as well as practical competence.

Values that apply in offline relationships may also be relevant in digital settings. Respect may concern how individuals communicate online; responsibility may concern the sharing of information or images; and concern for others may influence responses to online harm. Privacy and consent acquire particular significance where digital material can be copied and distributed rapidly. Digital citizenship cannot be reduced to personal values because it also involves knowledge, skills, opportunities and technological conditions. Digital environments nevertheless provide settings in which broader values may be interpreted and enacted.

Informed digital participation also depends on the ability to evaluate and integrate information. Incognito and Tarchi (2024) found, among 165 lower-secondary students, that the ability to judge an author’s competence was associated with integrating information across multiple texts after relevant control variables were considered. Their findings illustrate why a self-report item about checking whether information is trustworthy should not be interpreted as a direct measure of digital literacy or sourcing skill. It records a reported practice that is normatively relevant to digital responsibility, whereas demonstrated sourcing and intertextual integration require task-based assessment.

Similar caution is required when examining environmental responsibility, personal agency, relationships and financial decision-making. These domains involve competencies, knowledge, social circumstances and opportunities as well as values. Questions about caring for the environment, making independent decisions, respecting personal boundaries or managing money may capture value-related orientations, but they should not be interpreted as comprehensive measures of environmental literacy, autonomy, relationship competence or financial literacy.

Self-report surveys allow patterns of endorsement to be examined across relatively large groups. They can identify which principles are widely recognized, which questions produce greater variation and whether response patterns differ across demographic or educational categories. They record responses to specified questions, however, rather than directly observing conduct.

This limitation is especially relevant when questions concern socially approved qualities. Responses to sensitive or normatively charged questions may be influenced by perceptions of social acceptability (Tourangeau and Yan, 2007). Values questions are not necessarily sensitive in the same way as questions concerning illegal or highly stigmatized behavior, but respondents may recognize that some answers are more socially desirable than others. Socially desirable responding should therefore be acknowledged as a possible influence without being assumed to account for all favorable responses.

The interpretation of subgroup differences also requires attention to their practical magnitude. With large samples, statistically significant findings may arise even where the practical, real-life magnitude of the differences between groups are small and their response distributions overlap extensively. Effect sizes are therefore necessary alongside significance tests. This is particularly important where findings might otherwise be interpreted as evidence of demographic divisions or contrasting value systems.

Large comparative programs such as the European Values Study have generated extensive evidence concerning religion, family, morality, trust and social participation. Their standardized methods permit comparisons across countries and, through repeated waves, over time (Luijkx et al., 2021). This research demonstrates that values are situated within historical, cultural and institutional contexts rather than following a uniform pattern across societies.

The European Values Study, however, primarily concerns adults and includes domains associated with adult social, political and family life. Its findings provide important comparative context but cannot be transferred directly to students in the early secondary years. Research on children’s values demonstrates the importance of adapting language, presentation and social contexts to younger respondents (Döring et al., 2010), while adolescents’ own accounts indicate that values acquire meaning through experiences of identity, relationships and decision-making (Lewis-Smith et al., 2021).

A growing body of research has examined values directly among children and early adolescents. In a cross-cultural study involving 3,088 children aged 7–11 from six countries, Döring et al. (2015) found that children could differentiate among the motivational value orientations proposed by Schwartz and generally assigned greatest importance to self-transcendence values and least importance to self-enhancement values. A subsequent multicultural study involving 4,615 children aged 6–12 similarly found that children’s value systems increasingly approximated the theoretically expected structure during middle childhood (Daniel et al., 2023). Research using the Children’s Values Questionnaire has also shown that school-aged children can report multidimensional values and that older children display greater differentiation among the contexts in which values are applied (Fyffe and Hay, 2021).

These studies demonstrate that values can be investigated meaningfully among younger populations using developmentally appropriate instruments. They do not, however, provide direct benchmarks for the present surveys, which were not designed as measures of the complete Schwartz value structure and included a broader mixture of value endorsement, perceived experience, self-reported behavior, agency and scenario-based responses.

Research in Malta has examined values-related questions including religion, solidarity and social attachment among adults (Abela, 2004). Considerably less evidence has been generated directly from Maltese students during early adolescence. This is an important gap because students aged approximately 11–13 are navigating changing relationships, increasing autonomy and expanding participation in school, peer and digital environments, while remaining under-represented in large-scale comparative values research.

### 1.1 The present study

The present study addresses this gap through two cross-sectional surveys administered to Year 7 and Year 8 students in Malta. Schwartz’s theory was used as a sensitizing conceptual lens for defining personal values and for explaining why values should be distinguished from attitudes and behavior. The questionnaires were not designed to operationalize the complete Schwartz model, reproduce the European Values Study or estimate latent value dimensions. Instead, they examined value endorsement and adjacent response types through situations considered meaningful to students in the respective cohorts.

Because the questionnaires differed in content, the study does not test developmental change between Year 7 and Year 8. The cohorts were independent, and the instruments did not permit direct item-level comparison. Each survey was therefore analyzed in relation to the domains that it measured. Together, the datasets provide complementary, but not equivalent, accounts of value-related orientations and reported practices among students in adjacent school years.

The analyses also explored item-level differences by gender, school sector, country of birth and religious affiliation. Statistical significance was considered alongside effect size so that reliable but substantively small differences were not overinterpreted. These comparisons concern responses to individual items; they do not test whether demographic groups have equivalent or different latent value structures.

The following research questions guided the study:

1. What item-level patterns are evident in students’ value endorsement, normative attitudes, perceived experiences, self-reported behaviors, agency or self-belief, and scenario-based behavioral intentions?
2. How do the descriptive patterns differ across these operational item types, particularly between broad evaluative items and items concerning reported or intended action?
3. What demographic differences are observed in individual item responses, and what is their practical magnitude?

## 2 Materials and methods

### 2.1 Study design and participants

A questionnaire-based design was selected because the study sought a broad item-level overview of value endorsement and related responses across Malta’s three school sectors. The questionnaires enabled students to report what they regarded as important, their evaluations of particular social principles, how they perceived selected experiences, how they believed they usually behaved, their perceived capacity to act, and how they intended to respond in age-relevant scenarios. These response targets were analyzed separately rather than combined into construct scores.

Self-report data nevertheless record responses to specified questions rather than directly observed conduct. Responses to normatively charged items may also be influenced by perceptions of social desirability. The findings were therefore interpreted as reported values, perceptions, usual behaviors and intended responses rather than as objective measures of students’ behavior.

This quantitative, cross-sectional study examined values and related orientations among Year 7 and Year 8 students in Malta during the 2025–2026 academic year. Two separate questionnaires were administered to independent cohorts. Because the instruments differed in content, the cohorts were analyzed separately and were not treated as parallel forms or as longitudinal measures of value development.

The target population comprised students aged approximately 11–13 years attending State, Church and Independent schools. The national Year 7 population consisted of 4,664 students, of whom 718 provided valid responses (15.4% population coverage). The Year 8 population consisted of 4,589 students, of whom 615 provided valid responses (13.4% population coverage). These coverage figures describe the size of the achieved samples relative to the cohorts; they are not probability-sample response rates.

The Year 7 participants had a mean age of 11.29 years (SD = 0.47; range = 10–13). Boys accounted for 53.38% of valid gender responses and girls for 46.20%. Most respondents were born in Malta (89.03%) and identified as Christian (89.58%).

The Year 8 participants had a mean age of 12.32 years (SD = 0.48; range = 11–14). The gender distribution was approximately equal, with 49.43% identifying as boys and 49.10% as girls. Most were born in Malta (90.17%) and identified as Christian (88.82%).

School-sector representation differed from the corresponding national distributions. Church-school students were over-represented in both cohorts, particularly in Year 8, while State-school students were under-represented in both cohorts and Independent-school students were under-represented in Year 8. The samples were therefore not proportionally representative, and the small Year 8 Independent-school subgroup (*n* = 24) particularly limits sector comparisons.

### 2.2 Recruitment and procedure

Following ethical and institutional approval, all schools in the State, Church and Independent sectors with students in Year 7 and Year 8 were invited to participate through national educational channels. Heads of School acted as gatekeepers, distributing information sheets and parental opt-out forms and sharing the survey link with eligible students.

All students in the relevant year groups within participating schools were eligible unless their parents or guardians opted out or the students themselves declined participation. Parental or student opt-outs were not recorded. Only students whose parents or guardians did not opt out, and who themselves assented, responded to the survey. The achieved samples were therefore voluntary and school-mediated rather than probability samples. In addition, the analytical datasets did not retain school identifiers, so possible clustering of responses within schools could not be modeled.

The surveys were created and administered through Google Forms and were available in English and Maltese with survey questions and answer categories being visualized in both languages side by side to aid responses. Students completed them individually at school, generally using government-issued laptops, during an appropriate lesson and under teacher supervision. Completion took approximately 20–25 min.

Students were informed that participation was voluntary, that they could omit individual questions and that they could leave the survey before submission. Participation was not linked to assessment, school performance or disciplinary procedures. Moreover, no technical identifiers were collected.

### 2.3 Survey instruments

Two age-specific questionnaires were developed for the study. Their design was informed by the project’s conceptual framework and by wider international values research, including the European Values Study and World Values Survey. Schwartz’s theory served as a sensitizing conceptual lens rather than as a measurement model. The questionnaires were not designed to operationalize the complete Schwartz model, reproduce the European Values Study or estimate latent value dimensions. Instead, they examined value endorsement and adjacent response types through age-relevant situations considered meaningful to students in the respective cohorts. The complete questionnaires, response categories and coding procedures are provided in Supplementary materials S1, S2. Supplementary Table S1 classifies each closed item according to its principal response target.

The Year 7 questionnaire focused primarily on interpersonal and everyday contexts, including respect, tolerance, empathy, responsibility, helping others, friendship, cooperation, trust, honesty, environmental responsibility, fairness, safety, peaceful coexistence, physical activity and fair play. The Year 8 questionnaire addressed inclusion, equality, belonging, cultural identity, moral and religious values, digital responsibility, privacy, personal agency, decision-making, relationships, consent, personal boundaries, safety and financial responsibility.

Both questionnaires consisted mainly of closed-response items using importance, agreement and frequency scales, perceived-safety ratings and scenario-based multiple-choice questions. Ordinal questions generally used four or five response categories, such as “very important” to “not important,” “strongly agree” to “strongly disagree,” or “always” to “never.” Scenario-based questions presented an age-relevant situation and asked students to select the response most closely representing what they would usually do.

The surveys also included a limited number of open-ended questions. These responses are not included in the present quantitative analysis.

The two questionnaires did not contain identical items or measure equivalent domains. No direct item-level comparison was therefore made between Year 7 and Year 8, and differences between the cohorts were not interpreted as evidence of developmental change.

For interpretation, closed items were classified by their principal response target: (a) value endorsement, asking about the importance of a broad principle or goal; (b) normative attitude, asking whether a specific principle or judgment should be accepted; (c) perceived experience or context, asking how the respondent was treated, felt or experienced an environment; (d) self-reported behavior or practice, asking how often the respondent claimed to act in a particular way; (e) agency or self-belief, asking whether the respondent believed they could act, refuse or resist pressure; and (f) scenario-based behavioral intention, asking which response the student believed they would usually select in a described situation. These categories distinguish response targets; they are not latent scales. Some domains contained both broad evaluative items and more specific behavior-oriented or scenario-based items, but these were not designed as matched pairs and did not necessarily use identical response scales. The study therefore did not calculate an endorsement–enactment discrepancy or test whether individual students behaved inconsistently with values they endorsed. Comparisons across item types were descriptive only.

Questionnaire development followed an iterative review and pretesting process conducted by the authors. Draft items were reviewed by members of the author team with relevant expertise and by teachers and education officers familiar with the target age groups. The review considered the relevance and coverage of the content, developmental appropriateness, clarity of wording, readability and suitability of the response categories. The questionnaires were also informally piloted, and cognitive interviewing was used to examine how participants understood the questions and formulated their responses. Feedback from these processes informed revisions to the wording, organization and presentation of the items.

The questionnaires were developed in English and Maltese and underwent bilingual review to assess the clarity and conceptual equivalence of the two language versions. These procedures supported face and content validity, readability and age appropriateness, but they did not establish construct validity, reliability or measurement invariance. The questionnaires were not subjected to formal psychometric validation. Because they combined different response targets and were not constructed as unidimensional scales, internal-consistency coefficients and composite scores were not calculated. Analyses were conducted at item level, and the findings should be interpreted as responses to individual questions rather than scores on validated underlying value constructs.

### 2.4 Ethical considerations and data protection

The study was reviewed and approved by the University of Malta Faculty of Education Research Ethics Committee (approval reference: EDUC-2025-01341). Permissions were also obtained through the appropriate channels for State, Church and Independent schools.

The study used a parental opt-out procedure. Parents or guardians received information about the purpose and procedures of the research and could return a refusal form if they did not wish their child to participate. Student assent was obtained within Google Forms before the substantive questionnaire began.

No student names, email addresses or other direct or technical identifiers were collected. Survey responses were downloaded from Google Forms, encrypted and stored on password-protected systems accessible only to authorized members of the research team. Data were handled in accordance with the General Data Protection Regulation and University of Malta data-management requirements.

### 2.5 Data preparation

The Year 7 and Year 8 datasets were prepared and analyzed separately. Responses were checked for validity and completeness. Given that no identifying information was collected on respondents to preserve anonymity, no checks could be performed on duplicate submissions. No data weighting was applied. Since all questions were voluntary, submitting a survey with a response to at least one question was considered a valid response to the survey overall. Missing responses were retained as missing, and not imputed. Descriptive statistics for each question were calculated using the valid responses to each item. Consequently, denominators varied slightly across questions and analyses.

For subgroup analysis, gender comparisons were conducted principally between boys and girls because very few students selected “prefer not to say” (*n* = 3 for Year 7; *n* = 9 for Year 8). Country of birth was analyzed as born in Malta or born outside Malta. Religious affiliation was analyzed principally as Christian or another/no religious affiliation. School sector was retained as State, Church or Independent. Students who did not know their school sector were omitted from sector subgroup analyses (*n* = 43 for Year 7; *n* = 20 for Year 8). Respondents excluded from the subgroup analyses for gender (“prefer not to say”) or school sector (“do not know”) were included in other analyses for which they provided information.

The numerical direction of the ordinal scales differed across some questionnaire sections. The interpretation of rank-based tests therefore took account of the coding direction of the relevant item.

### 2.6 Statistical analysis

Descriptive statistics were used to summarize participant characteristics and item-response distributions. Frequencies and valid percentages were reported for categorical and ordinal variables, while means and standard deviations were used where appropriate.

For concise descriptive reporting, adjacent favorable response categories were sometimes combined, including “important” and “very important,” “agree” and “strongly agree,” and “often” and “always.” Inferential analyses used the original response categories rather than these collapsed groupings.

Because most outcomes were ordinal, non-parametric tests were used for exploratory demographic comparisons. Mann–Whitney U tests examined differences between two independent groups, including comparisons by gender, country of birth and religious affiliation. Kruskal–Wallis H tests were used for comparisons across State, Church and Independent schools. Where an omnibus school-sector test was statistically significant, adjusted post-hoc Dunn–Bonferroni pairwise comparisons were conducted.

Pearson’s chi-square tests of independence were used for single-choice nominal and scenario-based outcomes. The Year 8 bullying-and-exclusion item permitted multiple selections and was therefore reported descriptively rather than analyzed as if its response combinations were mutually exclusive categories.

All tests were two-sided, with statistical significance assessed at α = 0.05. Exact *p*-values were reported where available, with values below 0.001 presented as *p* < 0.001. Because numerous item-level comparisons were conducted and no composite constructs were tested, inferential findings are treated as exploratory: interpretation emphasizes effect size, recurrence across conceptually related items and overlap in response distributions rather than isolated *p*-values.

Effect sizes were reported alongside significance tests. For Mann–Whitney U tests, effect size r was calculated from the standardized test statistic. Epsilon-squared (ε^2^) was used for Kruskal–Wallis tests, and Cramér’s V was used for chi-square analyses. Effect sizes were interpreted using conventional thresholds. For Mann–Whitney U tests, effect sizes (*r*) of 0.10, 0.30, and 0.50 were interpreted as small, medium, and large, respectively. For Kruskal–Wallis tests, epsilon-squared (ε^2^) values of 0.01, 0.08, and 0.26 were interpreted as small, medium, and large effects, respectively. For Chi-square analyses, Cramer’s V was used to assess the strength of association. Values of 0.10, 0.30, and 0.50 were interpreted as indicating small, medium, and large effect sizes, respectively. Particular attention was given to effect sizes when interpreting subgroup differences, as statistically significant findings with very small effect sizes may have limited practical significance.

The analyses were conducted at item level. The questionnaires were not treated as validated unidimensional psychometric scales, and no overall or composite value scores were calculated. Consequently, the subgroup tests examine observed item-response distributions only; they do not establish construct-level group differences, measurement equivalence or the absence of such differences.

## 3 Results

### 3.1 Participant characteristics

The analytical samples comprised 718 Year 7 students and 615 Year 8 students. Their demographic characteristics and distribution across school sectors are presented in Table 1. The achieved samples covered 15.4 and 13.4% of the respective national cohorts, but participation was voluntary and school-mediated and sectoral representation differed from the national distributions. The results therefore describe the participating students and should not be interpreted as national prevalence estimates.

**Table 1:** Characteristics of the Year 7 and Year 8 samples.

Characteristic	Year 7	Year 8
National student population, *N*	4,664	4,589
Valid survey responses, *n*	718	615
Percentage of national population, % of *N*	15.4%	13.4%
Mean age, years	11.29	12.32
Boys, % of valid gender responses	53.38% *n* = 379	49.43% *n* = 301
Girls, % of valid gender responses	46.20% *n* = 328	49.10% *n* = 299
Born in Malta	89.03% *n* = 633	90.17% *n* = 541
Christian affiliation	89.58% *n* = 636	88.82% *n* = 540
State school	30.10% *n* = 214	33.82% *n* = 207
Church school	43.04% *n* = 306	58.99% *n* = 361
Independent school	20.82% *n* = 148	3.92% *n* = 24

Percentages are based on valid responses for each demographic variable. Thus, denominators vary due to item non-response.

The sectoral distribution of both samples differed from the corresponding national populations. Church-school students were over-represented, particularly in Year 8, while State-school students were under-represented in both cohorts and Independent-school students were under-represented in Year 8. The Year 8 Independent-school subgroup was especially small (*n* = 24), so apparent sector differences involving this group are unstable.

### 3.2 Year 7 findings

#### 3.2.1 Respect, tolerance and empathy

Year 7 students reported strong endorsement of respect and openness toward others. A total of 95.95% considered respecting other people important or very important, while 96.62% considered listening to different opinions important or very important. Most students also reported positive experiences of interpersonal respect: 81.49% stated that classmates treated them respectfully always or most of the time.

Acceptance of difference was also widely reported. Almost three-quarters of respondents (72.92%) selected the scores 4 or 5 on the five-point comfort scale (1-not important; 5-very important) when asked how comfortable they felt with classmates who were different from them. Empathic orientation was somewhat less uniform but remained positive, with 72.09% stating that they often or always tried to imagine how another person felt.

#### 3.2.2 Responsibility, rules and contribution

Responsibility received strong endorsement. Overall, 96.62% regarded doing their share of work at school or at home as important or very important. In addition, 88.34% agreed or strongly agreed that, when they did something wrong, they should admit it and try to make it right.

Students reported greater involvement in improving their immediate school environment than their wider neighborhood. A total of 63.68% stated that they often or always tried to make their school a better place, compared with 57.32% who reported doing so for their neighborhood.

Rules were viewed positively. Most respondents agreed or strongly agreed that rules help to keep people safe (91.93%) and ensure fair treatment (87.78%). Consistent rule-following was also widely reported, with 92.69% stating that they followed classroom or school rules always or most of the time.

#### 3.2.3 Friendship and cooperation

Friendship and cooperation were among the most strongly endorsed domains. Having trustworthy friends was considered important or very important by 98.02% of respondents, while 98.03% agreed or strongly agreed that they tried to be a good friend and help friends when needed.

Cooperative behavior was also common. A total of 90.30% reported helping classmates always or often during group work, and 88.14% stated that they often or always included others when playing or working together. When disagreements arose between friends, 77.46% selected talking and trying to resolve the disagreement together as their usual response.

#### 3.2.4 Honesty and trustworthiness

Truth-telling was considered important or very important by 96.60% of respondents. Most students agreed or strongly agreed that others could trust them to do what they promised (87.75%) and that they tried to be someone whom others could trust (94.79%).

Scenario-based responses also indicated strong endorsement of honesty. When asked what they would usually do after breaking something accidentally, 80.87% stated that they would tell the truth and try to repair the damage. Responses concerning the regular admission of mistakes were more mixed: 68.68% reported doing so always or often, while 25.00% selected “sometimes.”

#### 3.2.5 Environmental responsibility

Environmental responsibility was strongly endorsed. A total of 97.87% regarded caring for nature and the environment as important or very important. Most respondents agreed that they tried not to waste water or electricity (91.62%), and 76.81% reported recycling or reusing materials always or often. A further 85.62% agreed that young people could contribute to protecting the planet.

Responses to the scenario were more varied than responses concerning the general importance of environmental responsibility. When asked how they would respond to rubbish found outdoors in nature, 59.89% stated that they would pick it up and dispose of it, while 23.76% would tell someone.

#### 3.2.6 Fairness, safety and peaceful coexistence

Students generally reported feeling safe in their everyday environments. A total of 94.61% felt very or quite safe at school, 98.70% at home and 90.48% in their neighborhood.

Peaceful conflict resolution was also strongly supported. Overall, 88.81% agreed or strongly agreed that problems should be resolved without fighting, while 92.30% agreed that calm discussion was preferable to shouting or hitting.

Fair play and inclusion in sport received similarly positive responses. Physical activity was regarded as important or very important for health and wellbeing by 97.28% of respondents. A total of 92.01% agreed or strongly agreed that they tried to play fairly and follow the rules even when nobody was watching. In addition, 84.19% reported encouraging others always or often, and 86.32% reported including others so that everyone could participate.

Table 2 summarizes selected item-level responses across the principal Year 7 domains. These were chosen to represent both the consistently high endorsement of prosocial values and areas in which responses to behavior-oriented or scenario-based items were more varied.

**Table 2:** Selected item-level responses among Year 7 students.

Domain	Questionnaire item	Valid *n*	Favorable response categories	%
Respect	Considered respecting other people important	716	Important/very important	95.95
Respect for perspectives	Considered listening to different opinions important	710	Important/very important	96.62
Empathy	Tried to imagine how another person felt	713	Often/always	72.09
Responsibility	Considered doing one’s share at school or home important	711	Important/very important	96.62
Accountability	Agreed that wrongdoing should be admitted and put right	712	Agree/strongly agree	88.34
Friendship	Considered having trustworthy friends important	709	Important/very important	98.02
Cooperation	Helped classmates during group work	711	Often/always	90.30
Inclusion	Included others when playing or working together	708	Often/always	88.14
Honesty	Considered always telling the truth important	706	Important/very important	96.60
Accountability in practice	Admitted mistakes	712	Often/always	68.68
Environmental responsibility	Considered caring for nature and the environment important	706	Important/very important	97.87
Environmental action	Picked up rubbish found outdoors in nature	703	Selected response	59.89
Peaceful conflict resolution	Agreed that calm discussion was preferable to shouting or hitting	701	Agree/strongly agree	92.30
Fair play	Tried to play fairly and follow rules even when unobserved	701	Agree/strongly agree	92.01

Percentages are based on item-specific valid responses (valid *n*). Favorable categories were combined for descriptive presentation only. The table presents selected item-level responses and does not include every item in the Year 7 questionnaire.

### 3.3 Year 8 findings

#### 3.3.1 Inclusion, equality and belonging

Year 8 students reported strong support for inclusion and equal treatment. A total of 90.23% considered accepting people who were different important or very important. Equal treatment was supported by 94.07%, while 91.83% agreed that people should have equal opportunities regardless of their origins.

Reported responses to exclusion and bullying were generally supportive. Students most commonly stated that they would help the person affected, inform a teacher or trusted adult, or check on the person afterwards. Only a small minority indicated that they would take no action. Inviting an isolated classmate to participate was reported always or often by 56.79% of respondents. Standing up against unfair treatment was less consistent: 20.56% reported always doing so, 34.33% often and 31.67% sometimes.

Belonging was important to most respondents. A total of 89.28% regarded feeling part of their school or class as important or very important, while 92.92% agreed or strongly agreed that they felt proud to belong to their family or community. Knowledge of one’s own culture and family origins was regarded as important or very important by 87.15%. At the same time, 51.89% reported learning about other cultures or countries often or very often. Almost all respondents (94.88%) agreed that everyone should have an opportunity to express ideas and be heard.

#### 3.3.2 Moral and religious values

Kindness and forgiveness were considered important or very important by 96.06% of respondents. A total of 87.81% agreed or strongly agreed that they tried to consider what was right and wrong in their actions. Respect for people with different beliefs was endorsed by 90.08%, while 93.07% regarded hope or belief that circumstances could improve as important or very important.

Responses concerning forgiveness in practice were more varied. Although 68.38% stated that they always or often tried to forgive someone who had hurt their feelings, a substantial minority reported doing so less frequently.

#### 3.3.3 Digital responsibility

Digital responsibility received high levels of endorsement. Keeping personal information private online was considered important or very important by 96.01% of respondents. A total of 90.26% agreed or strongly agreed that they tried to communicate kindly and respectfully online.

Consent in image-sharing was also widely recognized: 77.17% reported always or often asking permission before posting or sharing photographs or videos of another person. When responding to online bullying, 38.21% stated that they would report or block the account, while 26.41% would tell a trusted adult. By contrast, 24.42% indicated that they would ignore the behavior and 10.13% would actively support the person targeted. Joining in was reported by only 0.83%.

Most respondents reported checking the trustworthiness of information before sharing it, with 80.04% selecting always or often. Screen-time self-regulation produced more varied responses: 69.54% agreed or strongly agreed that they exercised enough self-control to prevent screen use from interfering with sleep, schoolwork or family time; 22.35% were uncertain and 8.11% disagreed or strongly disagreed.

#### 3.3.4 Agency and autonomy

Students strongly endorsed having a voice in decisions affecting them. Having a say at home was considered important or very important by 92.66%, while 91.31% considered having a say at school important or very important.

A total of 83.70% agreed or strongly agreed that they could refuse something with which they were uncomfortable. Before agreeing to a request or commitment, 73.96% stated that they always or often sought further information. When faced with pressure from a friend to do something uncomfortable, 51.58% reported that they would refuse and explain their feelings, while 33.61% would suggest an alternative. Together, these two responses accounted for 85.19% of respondents.

Respect for the safe and fair choices of others was endorsed by 92.68%. In addition, 84.95% stated that they always or often tried not to exclude others and showed kindness toward people who were different.

#### 3.3.5 Relationships, consent and safety

Access to reliable information about growing up and relationships was regarded as important or very important by 96.64% of respondents. A total of 86.36% agreed or strongly agreed that they could refuse unwanted physical contact, while 76.57% reported always or often seeking permission before touching another person.

Responses to requests involving private images were generally protective. Half of respondents (50.34%) stated that they would refuse and would neither share nor retain the image, 20.97% would report or block the person or account, and 18.12% would tell a trusted adult. Only 1.01% reported that they would share such an image with friends.

Respect for differences in identity and feelings was endorsed by 91.95%. Help-seeking was also relatively common, with 67.23% reporting that they always or often asked a trusted adult questions about growing up.

#### 3.3.6 Financial responsibility

Financial responsibility was one of the most strongly endorsed Year 8 domains. Looking after money and keeping it safe was considered important or very important by 98.48% of respondents. Saving money for later was reported always or often by 83.84%.

When students wanted to buy something but did not have enough money, 57.96% reported that they would wait and save, 17.09% would select a less expensive option and 12.73% would ask a parent or carer for advice. Borrowing with a repayment plan was selected by 4.52%, while stealing was selected by 0.34%.

Most respondents (88.94%) agreed or strongly agreed that they could resist pressure from friends or advertising to purchase something unnecessary. A total of 86.19% reported always or often comparing prices or choices in order to use money wisely.

Table 3 summarizes selected item-level responses across the principal Year 8 domains. These were chosen to illustrate both the strong endorsement of inclusion, equality, digital responsibility, agency, personal boundaries and financial responsibility, and the more varied responses to items concerning social inclusion, moral courage and help-seeking.

**Table 3:** Selected item-level responses among Year 8 students.

Domain	Questionnaire Item	Valid *n*	Favorable response categories	%
Inclusion	Considered accepting people who are different important	604	Important/very important	90.23
Equality	Supported treating everyone fairly and equally	607	Favorable categories	94.07
Belonging	Considered feeling part of their school or class important	606	Important/very important	89.28
Inclusion in practice	Invited isolated classmates to join	604	Often/always	56.79
Moral courage	Stood up against unfair treatment	603	Often/always	54.89
Moral values	Considered kindness and forgiveness important	609	Important/very important	96.06
Ethical reflection	Tried to think about what was right and wrong	607	Agree/strongly agree	87.81
Digital privacy	Considered keeping personal information private online important	602	Important/very important	96.01
Online respect	Tried to communicate kindly and respectfully online	606	Agree/strongly agree	90.26
Digital consent	Sought permission before sharing another person’s image	600	Often/always	77.17
Information verification	Checked whether information was trustworthy before sharing it	601	Often/always	80.04
Agency	Agreed that they could say no when uncomfortable	601	Agree/strongly agree	83.70
Personal boundaries	Agreed that they could refuse unwanted physical contact	594	Agree/strongly agree	86.36
Help-seeking	Asked a trusted adult about questions concerning growing up	592	Often/always	67.23
Financial responsibility	Considered looking after money and keeping it safe important	591	Important/very important	98.48

Percentages are based on item-specific valid responses (valid *n*). Favorable categories were combined for descriptive presentation only. The table presents selected item-level responses and does not include every item in the Year 8 questionnaire.

### 3.4 Demographic differences

Exploratory item-level comparisons identified a number of statistically significant differences by gender, school sector and religious affiliation, while differences by country of birth were limited and did not form a consistent pattern. Reported effect sizes for the selected findings in Table 4 were small or very small. These analyses concern individual response distributions and should not be interpreted as comparisons of validated value scores.

**Table 4:** Selected exploratory item-level differences by demographic group and cohort.

Domain	Questionnaire item	Group difference	Cohort	*p*-value	Effect size
Empathy	How often do you try to imagine how someone else feels?	Girls > Boys	Year 7	*p* < 0.001	*r* = 0.15
Rule-following	How often do you try to follow the class or school rules?	Girls > Boys	Year 7	*p* = 0.01	*r* = 0.11
Improvement of school environment	How often do you try to make your school a better place (for example by helping or keeping it clean)?	Girls > Boys	Year 7	*p* < 0.001	*r* = 0.18
Inclusion	How often do you help your classmates when you work in a group?	Girls > Boys	Year 7	*p* < 0.001	*r* = 0.17
Inclusion	Do you agree or disagree? “I try to take turns and share when playing or doing activities.”	Girls > Boys	Year 7	*p* < 0.001	*r* = 0.12
Inclusion	How often do you include others when playing or working together?	Girls > Boys	Year 7	*p* < 0.001	*r* = 0.15
Trust	How important is it for you to have friends you can really trust?	Girls > Boys	Year 7	*p* < 0.001	*r* = 0.11
Trust	Do you agree or disagree?: “People can trust me to do what I promise.”	Girls > Boys	Year 7	*p* < 0.001	*r* = 0.13
Trust	Do you agree or disagree?: “I try to be someone others can trust.”	Girls > Boys	Year 7	*p* < 0.001	*r* = 0.14
Inclusion	Do you agree or disagree? “Everyone should have the chance to share their ideas and be listened to.”	Girls > Boys	Year 8	*p* < 0.001	*r* = 0.18
Inclusion	How often do you make sure not to leave people out and show kindness to those who are different from you?	Girls > Boys	Year 8	*p* = 0.002	*r* = 0.13
Online conduct	Do you agree or disagree?: “I try to be kind and respectful when I post or chat online.”	Girls > Boys	Year 8	*p* < 0.001	*r* = 0.12
Online conduct	How often do you ask the person first before you post or share their photo or video?	Girls > Boys	Year 8	*p* = 0.02	*r* = 0.10
Boundary setting	Do you agree or disagree: “I can say ‘no’ if I’m not OK with something.”	Girls > Boys	Year 8	*p* = 0.003	*r* = 0.12
Boundary setting	Do you agree or disagree?: “I can say ‘no’ to anything I do not want (like hugs or touching).”	Girls > Boys	Year 8	*p* = 0.01	*r* = 0.11
Rule-following	How often do you try to follow the class or school rules?	Church schools > Independent schools	Year 7	omnibus *p* = 0.02; adjusted *p* = 0.02	ε^2^ = 0.01
Rule-following	How often do you try to follow the class or school rules?	Christian > Other	Year 7	*p* = 0.03	*r* = 0.08
Responsibility	How important is it to do your share of the work at school or at home?	Christian > Other	Year 7	*p* = 0.03	*r* = 0.08
Trust	How important is it for you to have friends you can really trust?	Christian > Other	Year 7	*p* < 0.001	*r* = 0.12
Trust	Do you agree or disagree?: “I try to be someone others can trust.”	Christian > Other	Year 7	*p* < 0.001	*r* = 0.21
Online conduct	Do you agree or disagree?: “I try to be kind and respectful when I post or chat online.”	Christian > Other	Year 8	*p* = 0.02	*r* = 0.10
Boundary setting	Do you agree or disagree: “I can say ‘no’ if I’m not OK with something.”	Christian > Other	Year 8	*p* = 0.026	*r* = 0.09
Boundary setting	How often do you ask for more information before you say “yes” to something (like joining a group, sharing a photo, or taking part in an activity)?	Christian > Other	Year 8	*p* = 0.03	*r* = 0.09

Mann–Whitney U tests were used except for the school-sector comparison, which reports the Kruskal–Wallis omnibus effect (ε^2^) and the adjusted Dunn–Bonferroni Church–Independent pairwise *p*-value. The table presents selected recurring or illustrative item-level findings; it does not report latent value scores or tests of measurement equivalence.

Gender produced the most consistent recurring pattern. In Year 7, girls reported somewhat more favorable responses on items concerning empathy, rule-following, engagement in improving the school environment, inclusion and trust-related practices. In Year 8, girls reported somewhat more favorable responses on selected inclusion, online-conduct and boundary-setting items. The associated effects were small, and boys’ and girls’ distributions overlapped substantially.

School-sector differences were item-specific and should be interpreted especially cautiously because the sector groups were unequal and the Year 8 Independent-school subgroup was small. In Year 7, difference in reported rule-following was small (ε^2^ = 0.01).

Differences by country of birth were limited and did not show consistent divergence across the items examined. The much smaller group born outside Malta also restricts interpretation. The analysis does not establish equivalence between Malta-born and other respondents; it shows only that large, recurring item-level differences were not observed in these achieved samples.

Religious affiliation was associated with selected item-level differences. In Year 7, Christian respondents reported somewhat more favorable responses on selected responsibility, rule-following and trust items; in Year 8, differences appeared on selected online-conduct and agency items. Effect sizes ranged from very small to small. Because the comparison combined diverse minority-faith and non-religious respondents into one much smaller category, it cannot support claims about particular affiliations or value systems.

### 3.5 Summary of findings

The results also showed that favorable responses were less prevalent on some behavior-oriented and scenario-based items than on broader statements concerning the importance of a value. In Year 7, this pattern was evident in responses concerning the admission of mistakes and taking personal action when encountering rubbish outdoors. In Year 8, responses were less uniformly favorable when students were asked about inviting an isolated classmate to participate, standing up against unfair treatment, actively supporting a target of online bullying and regulating screen use. Because the relevant items differed in wording, response format and situational demands, these differences should not be interpreted as direct measures of inconsistency within individual students. They instead identify contexts in which reported responses were more varied than general statements of value endorsement.

## 4 Discussion

This study examined item-level value endorsement and related responses reported by Year 7 and Year 8 students in Malta. Value-importance and normative-attitude items generally elicited strong support for respect, fairness, cooperation, honesty, inclusion, responsibility and peaceful relationships. Responses were less uniformly favorable on selected self-reported behavior and scenario-based items involving socially demanding situations. Exploratory subgroup comparisons identified a recurring gender pattern, but effects were small and the analyses do not establish construct-level demographic differences.

### 4.1 Prosocial principles across distinct item types

The strongest descriptive pattern was the broad endorsement of prosocial principles on value-importance and normative-attitude items. Year 7 students responded particularly favorably to items concerning respect, trustworthy friendship, cooperation, honesty, environmental responsibility and peaceful conflict resolution. Year 8 students responded similarly to items concerning inclusion, equality, digital privacy, respectful online conduct, personal boundaries and financial responsibility. These findings concern the content of individual responses, not a validated prosocial-value scale.

The broad endorsement of prosocial principles is directionally consistent with research involving children in other national settings. Döring et al. (2015), studying children aged 7–11 across six countries, found that self-transcendence values concerned with the welfare of others were generally assigned greater importance than self-enhancement values. Research using other age-adapted instruments has similarly shown that children and early adolescents can identify multidimensional values concerning relationships, social responsibility and appropriate conduct (Fyffe and Hay, 2021). The prominence of respect, cooperation, fairness, trust and concern for others in the present findings therefore corresponds with a wider developmental literature in which socially oriented values are already meaningful during childhood.

This directional correspondence must be interpreted cautiously. The present questionnaires did not measure Schwartz’s value dimensions directly and included normative attitudes, perceived experiences, reported practices, agency or self-belief, and scenario-based intentions alongside value-importance items. The findings cannot therefore be compared numerically with established values scales or used to infer the internal organization of students’ value systems. Their more limited contribution lies in documenting how participating Maltese students responded to age-relevant questions across several clearly distinguished response types.

The favorable value-importance responses are consistent with research showing that values are meaningful during childhood and early adolescence. Although value priorities display both stability and change during these periods, younger adolescents can distinguish among values and relate them to everyday situations (Cieciuch et al., 2016; Daniel and Benish-Weisman, 2019; Vecchione et al., 2020). The present study adds item-level evidence from Malta, while its non-validated instruments preclude claims about the structure or development of value priorities.

This reinforces the importance of developmentally appropriate measurement. Research with children and adolescents has shown that values can be investigated meaningfully when they are presented through accessible language and recognizable contexts rather than through adult-oriented abstractions alone (Döring et al., 2010; Lewis-Smith et al., 2021). In the present study, honesty, responsibility and inclusion were examined not only as general principles but also through situations involving mistakes, classmates, peer pressure, image-sharing and money.

### 4.2 Values, peer relationships and social cohesion

The Year 7 findings were particularly strong in domains connected to everyday interpersonal life. Respect, friendship, cooperation, trustworthiness and peaceful conflict resolution were widely endorsed, and most students reported helping and including classmates.

These orientations are relevant to the relational dimension of social cohesion. Schiefer and van der Noll (2017) identify social relations, belonging and orientation toward the common good as recurring dimensions of cohesion. The present surveys did not measure cohesion directly, and students’ responses cannot establish that their schools or Maltese society are cohesive. However, respect, trust, cooperation and non-violent conflict resolution are among the principles that can support constructive social relationships.

The prominence of friendship is also consistent with the growing importance of peers during adolescence. Peer relationships provide contexts for recognition, belonging, cooperation and the negotiation of social norms (Steinberg and Morris, 2001; Smetana et al., 2006). Kindschi et al. (2019) found that adolescents’ value priorities and friendship networks may be connected through both selection and social influence. The present study cannot determine the direction of these relationships, but the strong endorsement of trust and helping suggests that students attached considerable importance to the moral quality of friendships.

Year 8 students also reported strong support for inclusion, equal treatment and belonging. These findings align with research linking school belonging to motivational, social–emotional, behavioral and academic outcomes (Korpershoek et al., 2020). They also support the view that inclusive environments depend on more than demographic diversity. Equitable participation, supportive relationships and recognition of difference are important to whether students experience belonging in practice (Nishina et al., 2019; Choi et al., 2025).

The results should nevertheless be interpreted cautiously. Approximately 9 in 10 respondents were Malta-born and Christian. The study therefore provides stronger evidence about majority-group endorsement of inclusion than about the lived experiences of students from migrant, minority-faith or non-religious backgrounds. Moreover, belonging to a family, school or community does not necessarily imply openness toward unfamiliar groups. Abela’s (2004) distinction between close-group solidarity and wider forms of solidarity remains relevant: strong attachment within familiar circles may coexist with either openness or exclusion beyond them.

### 4.3 Digital responsibility, agency and personal boundaries

The Year 8 findings extend the analysis into digital and relational contexts. Students responded favorably to items concerning privacy, respectful online communication and consent before sharing images. These are normatively relevant to digital responsibility, but they represent a mixture of value endorsement, normative attitude and self-reported practice rather than a single digital-responsibility construct.

This pattern is consistent with Choi’s (2016) account of digital citizenship as encompassing ethics, information literacy, participation and critical engagement rather than technical competence alone. Incognito and Tarchi (2024) further show that sourcing skills—particularly judging an author’s competence—are associated with lower-secondary students’ integration of information across texts. The present item asking whether students checked trustworthiness before sharing information is therefore best interpreted as a self-reported practice relevant to informed citizenship, not as demonstrated sourcing skill, intertextual integration or digital literacy.

At the same time, scenario-based responses to cyberbullying were less consistent. Reporting or blocking the account and telling a trusted adult were common intentions, but almost one-quarter selected ignoring the incident and relatively few selected direct support for the person targeted. Screen-time self-regulation was also less uniformly favorable than privacy or respectful communication. These distributions do not demonstrate a failure to enact a value: they show that a hypothetical response and a broad evaluative judgment are different outcomes, shaped by confidence, skills, perceived social costs and situational constraints.

Year 8 students also strongly endorsed voice, agency and personal boundaries. Most considered having a say at home and school important and reported confidence in refusing uncomfortable situations or unwanted physical contact. These findings are consistent with research describing adolescence as a period of increasing autonomy within changing family, peer and institutional relationships (Smetana et al., 2006).

The response patterns indicate that students often recognized responsible conduct in terms of informed choice, consent and self-protection. However, the agency statements measure self-belief, while the help-seeking item measures a self-reported practice. Neither can be inferred from value endorsement alone, and neither establishes how students would behave when a boundary is challenged in practice.

### 4.4 General endorsement and responses in specific situations

One of the clearest descriptive patterns was that broad value-importance and normative-attitude items frequently received more favorable responses than items asking about reported or intended action in particular situations. Year 7 students strongly endorsed truth-telling and environmental responsibility, while responses were more varied when they were asked how often they admitted mistakes or what they would do when encountering rubbish outdoors. Among Year 8 students, acceptance of difference and equal treatment were widely endorsed, while inviting an isolated classmate to participate and standing up against unfair treatment received less uniformly favorable responses. Privacy and respectful online conduct were also strongly supported, but scenario-based responses to online bullying were more varied.

This pattern should not be interpreted as a directly measured gap between endorsement and enactment. The questionnaire items were not constructed as matched pairs and differed in wording, response format and situational specificity. The general importance assigned to honesty, for example, is not directly equivalent to the reported frequency of admitting mistakes, while valuing environmental care is broader than choosing one particular response to rubbish found outdoors. The study therefore cannot determine whether individual students failed to act consistently with values they personally endorsed.

Nevertheless, the pattern is consistent with research showing that values can guide behavior without determining it automatically. Their expression may depend on the relevance of a value to the situation, the presence of competing motivations, perceived consequences, social expectations and the opportunities available for action (Bardi and Schwartz, 2003; Sagiv et al., 2017). A student may value inclusion but hesitate to challenge peers publicly, or may value honesty while fearing punishment or embarrassment after admitting wrongdoing. Similarly, recognizing that online harm is wrong does not necessarily mean that a student feels confident about intervening directly.

Longitudinal evidence also suggests that the relationship between values and behavior may be reciprocal. Values may influence behavior, while repeated actions and experiences may contribute to the development of value priorities over time (Vecchione et al., 2016). Associations have also been identified between children’s personal values and teacher-rated classroom behavior, although these relationships are neither simple nor uniform (Scholz-Kuhn et al., 2023). The present findings should therefore be understood as identifying situations in which the translation of general principles into action may require further investigation, rather than as demonstrating a stable discrepancy between what students value and how they behave.

The reliance on self-report adds a further limitation. Students reported what they regarded as important, what they usually did or how they believed they would respond to a scenario; the study did not observe their behavior directly. Responses to normatively approved questions may also be influenced by perceptions of social desirability (Tourangeau and Yan, 2007). At the same time, the strong recognition of prosocial principles remains meaningful. It indicates that many students possessed a normative foundation upon which educational efforts to develop judgment, confidence and practical strategies could build.

### 4.5 Demographic differences in item responses

Gender was the most consistent demographic correlate in the exploratory analyses, with girls tending to report somewhat more favorable responses on selected empathy, inclusion, digital-conduct and boundary-setting items. This pattern is directionally consistent with cross-cultural research in which girls assigned greater importance to self-transcendence and conservation values, while boys assigned greater importance to self-enhancement values (Döring et al., 2015). Research among Bahamian sixth-grade students has also identified gender differences in value priorities (Liu et al., 2007).

The comparison must remain cautious. The present study did not administer a standard Schwartz values instrument, did not calculate construct scores and did not test measurement invariance. The observed effects were small or very small and concern responses to individual items, not distinct male and female value systems. Gendered expectations, socialization and differences in socially desirable self-presentation may also contribute to the pattern.

Response distributions overlapped substantially across the demographic categories examined. This overlap is important descriptively, but it does not demonstrate that the groups have the same values or equivalent latent constructs. The unequal subgroup sizes, non-probability sampling and absence of validated, invariant measures preclude such a conclusion.

From the perspective of social cohesion, widespread favorable responses to respect, fairness, inclusion and peaceful relationships may indicate a degree of shared normative language among the participating students. They do not establish that students’ value systems are equivalent, that experiences of exclusion are absent, or that schools or Maltese society are cohesive.

The findings therefore caution against deficit-based assumptions about particular groups while also cautioning against claims of sameness. Educational responses can provisionally build on the principles widely endorsed in these samples while attending to differences in experience, opportunity and the practical demands of action.

### 4.6 Implications

The findings have implications for how the values of young adolescents are understood. The strong endorsement of respect, fairness, honesty, inclusion, responsibility and peaceful relationships challenges accounts that portray young people primarily in terms of moral decline or deficiency. The results instead suggest that many students already recognize a substantial body of prosocial principles. The relevant question is therefore not simply whether young adolescents possess values, but how these values are interpreted and applied across different contexts.

The more varied responses to behavior-oriented and scenario-based items also indicate that general endorsement and situated action place different demands on young people. Acting consistently with a value may require confidence, social judgment, emotional regulation and a willingness to accept possible interpersonal consequences. Intervening against unfair treatment, admitting a mistake or supporting someone targeted online may involve peer pressure, uncertainty or fear of social costs. Support should therefore extend beyond communicating desirable principles and should help young people develop practical strategies for responding when values conflict or when action is socially difficult.

This implication extends across the environments in which young people participate, including families, schools, peer groups, community organizations and digital spaces. Adults and institutions can strengthen the conditions under which prosocial values are more readily enacted by modeling respectful conduct, responding consistently to unfairness, providing trustworthy avenues for help-seeking and allowing young people meaningful opportunities to express their views. Such support should not assume that action depends solely on individual character, since behavior may also be shaped by relationships, institutional practices and the perceived safety of intervening.

The generally small demographic effects caution against approaches based on fixed assumptions about particular categories of students. They do not prove demographic equivalence. Broad initiatives may be organized around principles that received widespread support in these samples, while targeted support can address situations in which students experience uncertainty, pressure or unequal opportunities to act.

The digital findings reinforce the importance of treating online conduct as part of young people’s wider social and moral lives while distinguishing values from competence. favorable responses concerning privacy, consent and respectful communication provide a normative starting point. More varied responses to online bullying and screen-time regulation indicate areas for further study of bystander action, help-seeking, peer influence and demonstrated sourcing or self-regulation skills; they do not establish how students behave online.

These implications should remain proportionate to the study’s design. The surveys measured reported values, perceptions and intended or usual responses rather than directly observed behavior, and they did not evaluate particular programs or institutional practices. The findings therefore identify promising directions for support and policy rather than demonstrating the effectiveness of any specific intervention.

### 4.7 Strengths, limitations and future research

The study provides item-level quantitative evidence from 1,333 valid responses across two cohorts and includes participants from Malta’s three school sectors. The use of age-specific questions enabled students to respond to contexts relevant to their everyday experiences, and the reporting of effect sizes reduced the risk of interpreting statistical significance as substantive difference. These strengths concern sample size and contextual breadth, not national representativeness or psychometric validity.

Several limitations remain. The samples were voluntary, school-mediated and not proportionally representative by school sector. Church-school students were over-represented, particularly in Year 8, while the Year 8 Independent-school subgroup was small. Both cohorts were predominantly Malta-born and Christian, limiting interpretation of smaller demographic categories. Because school identifiers were not retained in the analytical datasets, possible clustering within schools could not be modeled. Findings should therefore be generalized neither to national prevalence nor to differences among specific minority groups.

The study relied on self-report and combined distinct response targets: value endorsement, normative attitudes, perceived experiences, self-reported behaviors, agency or self-belief, and scenario-based intentions. It did not observe behavior. The questionnaires were developed for this study and analyzed at item level rather than as validated psychometric scales. The cross-sectional design and non-equivalent cohort instruments also preclude conclusions about developmental change between Year 7 and Year 8.

Although the questionnaires underwent expert and practitioner review, informal piloting, cognitive interviewing, readability testing and bilingual review, they were not subjected to formal psychometric validation. These procedures provide evidence of face and content validity only; they do not establish construct validity, reliability or measurement invariance. Further research should develop or adopt theory-aligned scales, assess item comprehension systematically, test factor structure and reliability, and establish equivalence across demographic and linguistic groups before comparing latent value constructs.

Future research should use longitudinal designs and comparable, validated instruments to examine how value priorities and value-expressive behavior change across adolescence. Probability-based or carefully stratified recruitment, higher participation across sectors, and greater representation of migrant-background, minority-faith and non-religious students would strengthen population inference and subgroup analysis.

The relationship between endorsement and enactment also warrants direct investigation. Behavioral observations, vignette-based studies, diaries and multi-informant designs could clarify when young people act consistently with the values they report. Further work should also examine how school climate, student voice, curricular provision and teacher–student relationships are associated with values and behavior.

Digital contexts require particular attention. Future studies could investigate cyberbullying bystander behavior, image-sharing, misinformation, online peer pressure and the use of artificial intelligence. Participatory research with students would also help clarify how young people interpret conflicts between values and how they experience belonging, responsibility and pressure in everyday life.

## 5 Conclusion

The findings provide a broadly positive item-level account of participating young adolescents’ responses in Malta. Across both cohorts, value-importance and normative-attitude items generally elicited strong support for respect, fairness, cooperation, honesty, inclusion, responsibility and peaceful relationships. Year 8 students also responded favorably to selected items concerning digital privacy, consent, personal agency and financial responsibility.

At the same time, responses were less uniformly favorable on some items involving action in specific or socially demanding situations, including admitting mistakes, intervening against unfair treatment, responding to online harm and seeking support. Because the study did not use matched measures of value endorsement and behavior, these findings do not establish an individual-level endorsement–enactment gap. They instead identify contexts in which the application of broadly recognized principles may be more demanding and may require additional support.

Observed demographic effects were generally small or very small, and response distributions overlapped substantially. This supports a cautious description of convergence on many individual items within the achieved samples, but it does not demonstrate that demographic groups share the same latent values. The practical implication is correspondingly modest: education can build on principles that were widely endorsed here while helping students develop the judgment, confidence and skills required in demanding interpersonal and digital situations.

## Data Availability

The raw data supporting the conclusions of this article will be made available by the authors, without undue reservation.
