# Supplementary material for: Values and value-related responses in early adolescence: evidence from two large student surveys in Malta
Source: Front Psychol. 2026 Sep 17;17:1923199. doi: 10.3389/fpsyg.2026.1923199 (PMC13627365; doi:10.3389/fpsyg.2026.1923199)
Supplement: Supplementary file 1 [file Supplementary_file_1.docx]

Supplementary Material

# 1 Supplementary survey instruments

**Supplementary File 1.** Bilingual Year 7 survey instrument used in the study.

**Supplementary File 2.** Bilingual Year 8 survey instrument used in the study.
